# Low physical activity and depression are the prominent predictive factors for falling in older adults: the Birjand Longitudinal Aging Study (BLAS)

**DOI:** 10.1186/s12877-023-04469-x

**Published:** 2023-11-20

**Authors:** Sara Mortazavi, Ahmad Delbari, Mohsen Vahedi, Reza Fadayevatan, Mitra Moodi, Hossein Fakhrzadeh, Masoumeh Khorashadizadeh, Ameneh Sobhani, Moloud Payab, Mahbube Ebrahimpur, Hanieh-Sadat Ejtahed, Farshad Sharifi

**Affiliations:** 1 Department of Gerontology, University of Social Welfare and Rehabilitation Sciences, Tehran, Iran; 2 Iranian Research Center on Ageing, University of Social Welfare and Rehabilitation Sciences, Tehran, Iran; 3 Department of Biostatistics and Epidemiology, Substance Abuse and Dependence Research Center, University of Social Welfare and Rehabilitation Sciences, Tehran, Iran; 4 Social Determinants of Health Research Center, Birjand University of Medical Sciences, Birjand, Iran; 5 Elderly Health Research Center, Endocrinology and Metabolism Population Sciences Institute, Tehran University of Medical Sciences, Tehran, Iran; 6 Non-communicable Disease Research Center, Endocrinology and Metabolism Population Sciences Institute, Tehran University of Medical Sciences, Tehran, Iran; 7 Endocrinology and Metabolism Research Center, Endocrinology and Metabolism Clinical Sciences Institute, Tehran University of Medical Sciences, Tehran, Iran; 8 Obesity and Eating Habits Research Center, Endocrinology and Metabolism Clinical Sciences Institute, Tehran University of Medical Sciences, Tehran, Iran

**Keywords:** Aging, Older adults, Fall risk factors, Incidence, Physical activity, Depression

## Abstract

**Background:**

Falling in the older adults has many irreparable consequences, including hospitalization to long-term care centers and loss of independence, depression and social isolation, financial burden, and death. The present study was conducted to estimate the incidence of falls and their associated factors among community-dwelling older adults.

**Methods:**

This program is a population-based prospective cohort study (≥ 60 years) in Birjand City from 2019 to 2020. A total of 1418 participants were included in the study, and 1344 participants were analyzed according to the inclusion criteria. Thirty-nine risk factors were evaluated. Basic information included demographic information, lifestyle factors, general health and medical history, and mental and functional health.

**Result:**

The incidence of falls among community-dwelling older adults in the previous approximately 24 months in the present study was 9.26% in women and 2.65% in men. In the multiple Cox proportional regression model based on fall risk factors, there was a strong significant relationship between male sex (HR = 0.37, CI = 0.21 to 0.64), being physically active (HR = 0.59, CI = 0.36 to 0.96), moderate-to-severe depression (HR = 2.97, CI = 1.47 to 6.01), severe depression (HR = 3.26, CI = 1.24 to 8.54), and high risk of falls according to the TUG test (HR = 1.73, CI = 1.10 to 2.72).

**Conclusions:**

Inactivity and depression were recognized as important factors in falls in older adults. It is recommended for older adults to have an active lifestyle to prevent falls and to prioritize the diagnosis and treatment of depression in older adults. Women as a group at higher risk should be considered in prevention programs. In addition, the use of the TUG test to identify high-risk older adults should be considered.

**Supplementary Information:**

The online version contains supplementary material available at 10.1186/s12877-023-04469-x.

## Background

Falling is one of the most significant risks for older adults, leading to various severe consequences, such as hospitalization, long-term care center admissions, loss of independence, depression, fractures, financial burden, and even death [1, 2]. The medical cost of falls in the older adults is estimated at 50 billion dollars one year [3]. About 30,000 elderly Americans died in 2016 as a result of falls [4]. Falls have been the cause of about 50% reduction (4.6 years reduction) in quality-adjusted life years (QALYs) in American older adults [5].

Identifying the causative factors and implementing appropriate therapeutic interventions can be effective in preventing falls in older adults. Several studies have highlighted factors such as old age, gender (being female), reduced physical activity, fear of falling, vitamin D deficiency, sarcopenia, frailty, arthritis, polypharmacy, multimorbidity, depression, diabetes, and home hazards as significant risk factors for falls [6–8]. Among these, it has been determined that the older adults who are less active are 39% more likely to recurrent falls. And depressed older adults have 1.91 times more chance of falling [9, 10].

The risk factors for falls among the older adults in Iran have been reported to be distinct [11]. A study examining fall risk factors across twelve European countries revealed a significant difference in fall rates, primarily attributed to variations in the prevalence of intrinsic risk factors among them. Hence, when developing preventive programs, it is crucial to consider the differences in intrinsic risk factors between countries and tailor preventive planning according to each country’s specific priorities [12].

While numerous studies have been conducted to identify the risk factors for falls in older adults, it should be acknowledged that inherent cultural, social, economic, and environmental differences exist across societies. As a result, the same interventions cannot be universally applied to all societies, and the risk factors for falls may vary. To date, there has been no large-scale prospective study conducted in Iran that investigates falls in a significant number of community-dwelling older people, encompassing a wide range of risk factors [11].

Given the increasing older adults population in Iran and the detrimental impact of falls on health, this study was designed and conducted to determine the incidence of falls among Iranian older adults. Understanding the risk factors influencing falls among the older adults living in society is crucial for implementing necessary preventive measures. Therefore, this prospective cohort study aimed to investigate the risk factors associated with falls among community-dwelling older adults in Birjand City.

## Methods

This study presents the results of analyzing data obtained from the Birjand Longitudinal Aging Study (BLAS). The BLAS is an ongoing cohort study that focuses on community-dwelling older adults aged 60 years and above residing in the city of Birjand, which is located in the eastern part of Iran and serves as the capital of North Khorasan province. The primary objective of the BLAS is to estimate the incidence rate and identify risk factors associated with geriatric syndromes, including falls, among older adults. To achieve this, the study follows up with participants through annual telephone interviews conducted with both the individuals themselves and their informants. These interviews aim to gather information on outcomes of interest such as falls, hospital admissions, and deaths. To ensure the accuracy and validity of the collected data, the study employs several verification methods. The information obtained from the interviews is cross-verified through checks of the participants’ medical records, hospital information systems, national death registry data, and data from the basic insurance system.

By utilizing these comprehensive approaches to data collection, the study aims to provide reliable insights into the incidence of falls and other geriatric syndromes among community-dwelling older adults in Birjand. Through the analysis of these data, important risk factors associated with falls can be identified, contributing to the development of effective preventive strategies and interventions for this population.

We employed a multistage stratified cluster random sampling approach to select participants for the BLAS study. The sampling process was guided by geographic information systems (GIS). Initially, we identified seventy clusters based on zip codes, and within each cluster, twenty-four households were chosen using a random sampling method. To be eligible for the study, individuals had to meet certain criteria: they needed to be older adults aged 60 years or older who had been residing in Birjand city for at least several months prior and were capable of effective communication. Subjects with severe dementia, as determined by an Abbreviated Mental Test Score of less than three, were excluded from participation.

The baseline assessment data from the BLAS were utilized in this study. The data were collected between October 2018 and May 2019. Eight trained researchers conducted face-to-face interviews with the participants, including one of the confidential informants. However, each domain’s data were collected by a single researcher. An online software called Digit was employed for data gathering. This software has the capability to define validated values and prevent incomplete form filling. Further information regarding the study design, data collection, and measures in the BLAS can be found elsewhere [13, 14]. The BLAS obtained ethical approval from the Ethics Committee of Social Welfare and Rehabilitation Sciences (Ethical Code: IR.USWR.REC.1398.090).

### Data collection and definition of variables

The data were collected by trained researchers through comprehensive interviews with the participants and one of their informants. The interviews involved the use of several questionnaires, which included gathering demographic information such as age, sex, number of years of education, job status, marital status, and living arrangement. Additionally, the questionnaires covered topics such as smoking habits, self-reported health status (including urinary incontinence, hearing loss, visual impairment, chronic pain), and history of diseases approved by physicians (such as cancers, diabetes mellitus, thyroid dysfunction, chronic obstructive pulmonary disease, hypertension, congestive heart failure, cerebrovascular accident, coronary artery diseases, osteoporosis, osteoarthritis, and Parkinson’s disease).

The participants were followed every 12 months from the baseline data gathering through call interviews. During these interviews, the participants and one of their close informants were asked if they had experienced any falling accidents, either with or without injury, since the last evaluation. If falls were reported, additional information was collected regarding the number of falls, the timing of the falls, and the causes behind them. We also inquired about other health outcomes, including hospital admissions. In cases in which participants had passed away during the follow-up period, we obtained information from their relatives regarding the time and location of death, as well as the cause of death. For cases involving hospital admissions, we accessed the medical records from three hospitals in Birjand to determine the cause of admission and its relation to falls. Verification of deceased individuals and their time of death was conducted by cross-referencing the death registry of the health system. In this study, several biological risk factors were investigated, including inherent factors of the human body and health conditions.

Demographic information, such as age, sex, marital status, education years, and living arrangement, was gathered by asking the participants and cross-checking with their national identity cards. Height and weight were measured without shoes and with minimal clothing, with precision to the nearest 0.5 centimeters and kilograms, respectively. Calf circumference was measured on both legs at their largest using a tape measure. The arm circumference of both arms was measured at the midpoint between the olecranon (elbow tip) and the elbow. Waist circumference was measured just above the iliac crests on both sides. Body mass index (BMI) was calculated based on height and weight. All other anthropometric measurements were conducted with a precision of nearest to 0.5 centimeters.

Hand grip strength was measured using a digital hand dynamometer (Saehan DHD-1 Digital Hand Dynamometer, Soul, Korea). Six measurements were taken three times for each hand, and the maximum grip strength recorded was considered for further analysis.

Participants were asked to walk a distance of 4.57 m three times at their normal speed. The two fastest recorded times were noted, and the shortest time among them was considered for the calculation of gait speed. A gait speed less than 0.8 m per second was considered low gait speed.

The Mini-Mental State Examination (MMSE) is a widely used test that assesses cognitive function across six domains. Its primary purpose is to identify cognitive disorders and determine the severity of dementia. The MMSE assigns a maximum score of 30, with higher scores indicating better cognitive condition [15]. Foroughan et al. validated the Persian version of the MMSE. They established a cutoff point of 21, which was deemed appropriate for classifying participants’ cognitive status. In their study, this cutoff point demonstrated a sensitivity of 90% and specificity of 84%. Sensitivity refers to the percentage of individuals with cognitive impairment correctly identified by the test, while specificity indicates the percentage of individuals without cognitive impairment correctly classified as such [16].

We used subjective sleep quality question (question 9) from the Pittsburgh Sleep Quality Index to assess sleep quality. The validity of this questionnaire was confirmed in a study in Iran [17].

The assessment for depression was conducted using the 9-item version of the Patient Health Questionnaire (PHQ-9). This instrument provides scores ranging from zero to 27. A lower score indicates a better mood, while a higher score is considered indicative of a severely depressed mood. In the study by Dadfar et al. (2018), specific cutoff points were used to classify individuals into different categories: zero to four indicated a normal mood, five to nine represented minimum depression, 10 to 14 denoted mild depression, 15 to 19 signified moderate depression, and a score of 20 or above indicated severe depression. Dadfar et al. performed a psychometric evaluation of this tool to assess its reliability and validity [18].

The Time Up and Go (TUG) test involves standing up from a chair without a handle, which has a sitting height of 45 cm, walking a distance of three meters as quickly as possible, turning around, and then sitting back down on the chair. In the context of older adults, completing the TUG test within 12 s or less is considered a low risk of falling, while a TUG time exceeding 12 s indicates a high risk of falling [19]. A study conducted on Iranian older adults demonstrated that the TUG test has high validity in assessing their fall risk [20].

We utilized the Short Physical Performance Battery (SPPB) to evaluate the physical health performance of the participants. The battery consists of three tests: sitting and standing up from a chair five times with hands folded on the chest, measuring walking speed over a distance of 2.44 m, and assessing balance through three leg positions—full tandem, half tandem, and side by side stand [21].

To construct a wealth index, we initially selected assets that could differentiate between relatively affluent and economically disadvantaged populations. These assets included ownership of a refrigerator, vacuum cleaner, microwave oven, washing machine, dish machine, water purifier, LED television, smartphone, automobile, and house. To calculate the wealth index, we transformed all variables into dichotomous variables. Subsequently, we conducted principal component analysis (PCA) on these variables. The first factor derived from PCA was then designated as the wealth index. In addition, we utilized the quintile of the wealth index as one of the variables in our regression models. This allowed us to examine how the wealth index category influenced other variables of interest [22].

A validated questionnaire was used to obtain the participants’ drug history, which included inquiries to both the participants and their informants regarding medications taken for at least one month within the last 6 months. Over-the-counter medications, vitamins, and supplements used by the participants during this period were also considered. The medications consumed by the participants were cross-checked with the insurance claim data. Polypharmacy was defined as the use of five or more drugs.

To assess the level of physical activity, participants were asked about their activities during the previous week. Seniors who reported no activities at all were classified as the inactive group, while those who engaged in physical activity for one or more days during the last week were categorized as the active group.

The Mini Nutritional Assessment (MNA) tool was utilized to assess the nutritional status of the participants. The MNA comprises four components: anthropometric characteristics, general characteristics, nutrition assessment, and health assessment. Using the MNA, participants were classified into three groups: normal nourished (MNA score ≥ 24), at risk of malnutrition (17 < MNA score < 24), and malnourished (MNA score ≤ 17) [23]. Previous validation studies in the Persian language have demonstrated the good psychometric properties of this instrument [24].

The ability to perform activities of daily living (ADL) and instrumental activities of daily living (IADL) was evaluated using Barthel’s Index and Lawton’s instrumental activity of daily living, respectively. Barthel’s Index measures ten self-care skills essential for independent living, including feeding, grooming, bathing, dressing and undressing, using a toilet, controlling bladder and bowel movements, transferring from wheelchair to bed and vice versa, walking on level surfaces, and ascending and descending stairs [25]. The Iranian version of Barthel’s Index has demonstrated high reliability and validity [26]. Lawton’s instrument assesses eight complex skills necessary for independent living, such as using the telephone, shopping, meal preparation, housekeeping, laundry, medication management, financial management, and transportation [27]. The Persian version of this tool has been reported to have sufficient reliability and validity by Mirzadeh [28].

### Follow-up protocol

Fall accidents were recorded annually through phone interviews with the participants and their informant relations. We developed an expert-validated fall questionnaire that asks about the occurrence of falls. If a fall was reported, we inquired about the number of falls within the interval between two interviews. Additionally, we asked about the time and place of the falls (indoor or outdoor), as well as any complications resulting from the falls. We also investigated whether falls led to hospital admission by analyzing hospital information records. The results obtained from the interviews were cross-checked with the data from the hospital records.

### Statistical analysis

The characteristics of the participants were analyzed using the mean and standard deviation for continuous variables. Categorical variables were summarized using crude frequency and percentage. To compare the faller groups and nonfaller groups, independent t tests were employed for continuous data, and chi-square or Fisher’s exact tests were used for categorical data. The frequency of falls was adjusted according to age, sex, and the weighted population frequency of Birjand. We also standardized the prevalence based on the World Health Organization population from 2000 to 2025 using survey analysis techniques. To identify the risk factors associated with falls, univariate logistic regression models were applied individually for each factor that had demonstrated an association with falls in the literature review. In the multiple backward approach model, only the variables with a p value of association less than 0.1 were retained. Additionally, we utilized Cox proportional regression models to analyze the association between the time (from enrollment to study to first time falling) to fall (self-reported by participants or their informants) and other risk factors. Sensitivity analyses were conducted to account for subjects lost to follow-up. In one scenario, we assumed that all lost subjects experienced a fall event, while in another scenario, we assumed that none of the lost subjects had a fall. The point estimates from the alternative scenarios were compared to the 95% confidence interval of the main scenario. Furthermore, considering death as a competing risk for falls, we repeated our analysis and calculated subhazard ratios. All statistical analyses were performed using Stata version 14 (Texas, USA). A two-tailed test was used, and a significance level of P < 0.05 was considered statistically significant.

**Fig. 1:**
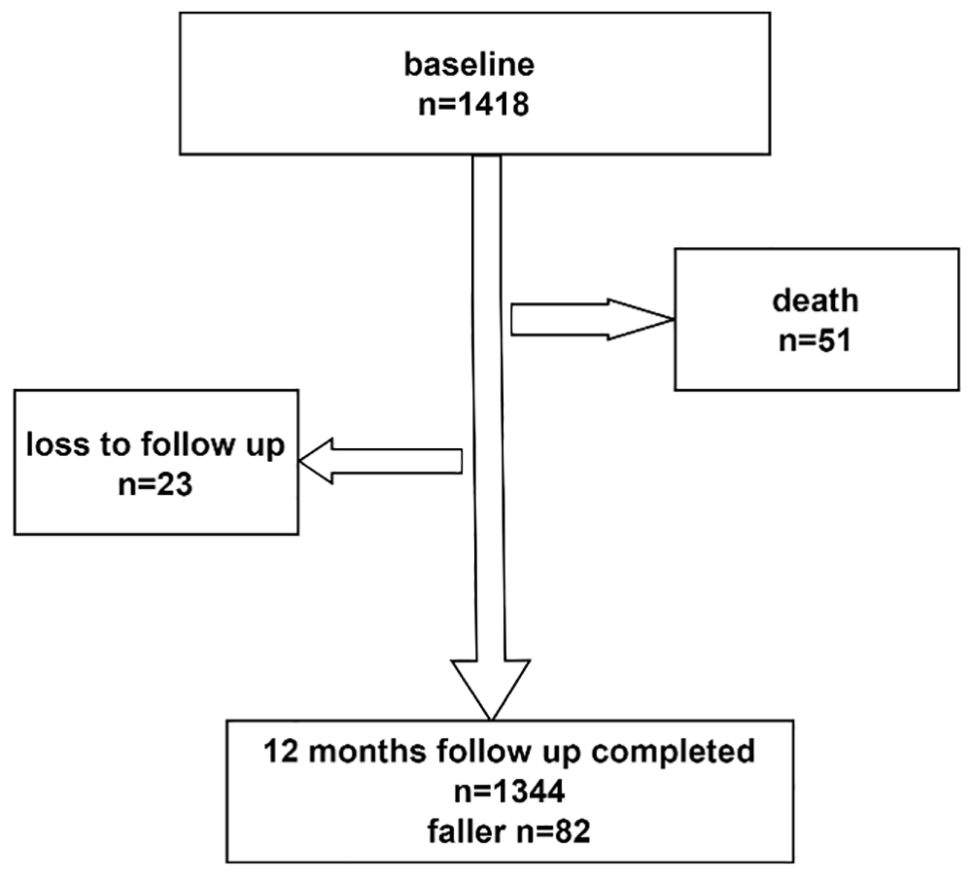
Flow chart of the study design in BLAS, 2018–2019

## Result

At the beginning of the BLAS study, 1418 individuals aged 60 and older were included. During the follow-up period, 51 participants passed away, 23 subjects were lost to follow-up, and 1344 subjects completed the study (Fig. 1). The maximum duration of the follow-up period was 819 days, with a median of 655 days and an interquartile range of 152 to 711 days. Among the total participants, 82 (6.10%) individuals (65 [9.26%] women and 17 [2.65%] men) reported experiencing falls, resulting in a total of 119 fall events. None of the falls resulted in hospital admissions. Of these, 63 subjects had one fall experience, 21 subjects had two falls, two subjects had three falls, two subjects had four falls, and three subjects had six falls. The incidence rate of falls was calculated as 4.68 per 100 person-years. Additionally, out of the total participants, 735 (51.8%) were women. The mean age of the participants was 69.72 years, with a standard deviation of 7.56 and a range of 60 to 96 years. Furthermore, 43.12% of the participants were illiterate, 78.2% were married, 11.25% lived alone, and 77.34% were housekeepers or retired (Table 1).

**Table 1:** Basic characteristics of the participants

Variables		Non-fallers n (%)	Fallers n (%)	Total (n)	P-Value
Age Group	60–69 years	738 (58.5)	39 (47.6)	777	0.131
	70–79 years	384 (30.4)	30 (36.6)	414	
	80 + years	140 (11.1)	13 (15.9)	153	
Sex (Female)		637 (50.5)	65 (79.3)	1344	< 0.001
Body mass index (BMI), (kg/m2)	Ideal body (18.5–24.9)	453 (35.9)	21 (25.6)	474	0.110
	Low body (≤ 18.5)	60 (4.8)	3 (3.7)	63	
	Overweight (25–30)	487 (38.6)	33 (40.2)	520	
	Obese (≥ 30)	261 (20.7)	25 (30.5)	286	
Low gait speed (< 0.8 m/s)		279 (22.1)	5 (6.1)	1344	< 0.001
Low grip strength (kg/cm2), (Female < 16) & (Male < 27)		502 (39.8)	42 (51.2)	1344	0.041
Calf circumference (cm), Mean ± SD		34.6 ± 4.7	35.2 ± 4.4	1343	0.214
Mid-arm circumference (cm), Mean ± SD		28.5 ± 3.7	29.4 ± 3.7	1343	0.045
Waist circumference (cm), Mean ± SD		95.2 ± 11.8	98.4 ± 11.7	1343	0.018
Urinary incontinence		150 (11.9)	12 (14.6)	1343	0.461
Cancer		23 (1.8)	3 (3.7)	1335	0.247
Hearing loss		126 (10.0)	10 (12.4)	1338	0.503
Visual impairment		324 (25.8)	24 (29.3)	1336	0.493
Diabetes mellitus		309 (24.5)	23 (28.1)	1343	0.471
Thyroid		70 (5.6)	6 (7.3)	1343	0.502
Chronic obstructive pulmonary disease		14 (1.1)	1 (1.2)	1338	0.930
Hypertension		537 (42.6)	40 (48.8)	1343	0.301
Congestive heart failure		146 (11.6)	11 (13.4)	1343	0.616
Cerebrovascular Accident		53 (4.2)	6 (7.4)	1340	0.174
Acute Myocardial Infarction		71 (5.7)	4 (4.9)	1325	0.752
Having Pain		957 (76.0)	69 (84.2)	1342	0.090
Osteoporosis		135 (10.8)	14 (17.5)	1334	0.064
Osteoarthritis		234 (18.6)	24 (29.3)	1338	0.018
Parkinson		9 (0.7)	0(0.0)	1343	0.443
Cognitive function (MMSE)	Normal cognitive	794 (63.3)	48 (59.3)	842	0.208
	Mild cognitive impairment	360 (28.7)	22 (27.2)	382	
	Dementia	100(8.0)	11(13.6)	111	
Depression (PHQ-9 score)	Normal mood (0–5)	684 (54.4)	31 (37.8)	715	< 0.001
	Mild depression (5–9)	344 (27.4)	19 (23.2)	363	
	Moderate depression (10–14)	159 (12.6)	16 (19.5)	175	
	Moderately-severe depression (15–19)	53 (4.2)	11 (13.4)	64	
	Severe Depressed (20–27)	17 (1.4)	5 (6.1)	22	
Sleep Quality	Very good	97 (7.7)	5 (6.1)	102	0.022
	Relative good	874 (69.3)	51 (62.2)	925	
	Relative poor	263 (20.9)	20 (24.4)	283	
	Very poor	27 (2.1)	6 (7.3)	33	
Physical activity, (Totally inactive)		583 (46.3)	55 (67.1)	1342	< 0.001
Impair Balance (TUG), High risk (> 12 s)		321 (25.4)	38 (46.3)	1344	< 0.001
SPPB score	Low (0–2)	37 (2.9)	6 (7.3)	43	< 0.001
	Intermediate (3–9)	372 (29.5)	35 (42.7)	407	
	Robust (10–12)	852 (67.6)	41 (50.0)	893	
Smoker		112 (8.9)	3 (3.7)	1343	0.101
Polypharmacy		186 (14.7)	19 (23.2)	1344	0.040
Nutrition status (MNA score)	Well nutrition (≥ 24)	945 (74.9)	49 (59.8)	994	< 0.001
	At risk of malnutrition (17–24)	306 (24.3)	31 (37.8)	337	
	Malnutrition (≤ 17)	10 (0.8)	2 (2.4)	12	
Dependency in ADL (Barthel’s Index 0–95 score)		380 (30.1)	35 (42.7)	1343	0.017
Dependency in IADL (Lawton’s IADL 0–7 score)		1002 (79.5)	66 (80.5)	1343	0.823
Marital status (Married)		1,048 (83.1)	59 (72.0)	1343	0.010
Job status	Retired	484 (38.4)	22 (26.8)	506	< 0.001
	Employed	161 (12.8)	2 (2.4)	163	
	Housekeeper	555 (44.0)	55 (67.1)	610	
	Unemployed	62 (4.9)	3 (3.7)	65	
Education years	Illiterate	551 (43.7)	43 (52.4)	594	0.252
	Primary school	405 (32.1)	23 (28.1)	428	
	High school	196 (15.5)	13 (15.9)	209	
	Academic	110 (8.7)	3 (3.7)	113	
Living arrangement (Live alone)		141 (11.2)	11 (13.4)	1343	0.536
Wealth Index (quintile)	First (Poorest)	238 (18.9)	18 (22.0)	256	0.576
	second	250 (19.8)	17 (20.7)	267	
	third	248 (19.7)	18 (22.0)	266	
	fourth	266 (21.1)	11 (13.4)	277	
	fifth (Richest)	259 (20.5)	18 (22.0)	277	

MMSE = Mini-Mental State Examination, PHQ-9 = Patient Health Questionnaire-9, TUG = Timed up and go, SPPB = Short Performance Physical Battery, MNA = Mini Nutritional Assessment, ADL = Activities of Daily Living, IADL = Instrumental Activities of Daily Living

MMSE scoring:

• Normal cognitive (Illiterate ≥ 19, Primary school ≥ 24, High school ≥ 27, Academic ≥ 28)

• Mild cognitive impairment (Illiterate = ≥ 14 MMSE score < 19, Primary school = ≥ 20 MMSE score < 24, High school = 24 ≥ MMSE score < 27, Academic = ≥ 25 MMSE score < 28)

• Dementia (Illiterate < 14, Primary school < 20, High school < 24, Academic < 25)

In the univariate logistic regression models, several variables were found to be significant risk factors for falls, including age, sex, obesity, increase in mid-arm circumference and waist circumference, low gait speed, osteoarthritis, depression, very poor sleep quality, low grip strength, high risk of TUG, polypharmacy, at-risk malnutrition status according to MNA, and being a housekeeper. Conversely, being physically active, increased SPPB score, increased scores of ADL and IADL, and being married were found to have a preventive role (Table 2).

**Table 2:** Associated between fall incidence and its risk factors in univariate and multiple logistic regression models

Variables		OR	95% CI OR	P-Value
**A univariate logistic regression model**				
Age (Each year increase)		1.03	1.00–1.06	**0.034**
Sex (Male/Female)		0.26	0.15–0.45	**< 0.001**
Body mass index (BMI), (kg/m2)	Ideal body (18.5–25)	Reference group		
	Low body (≤ 18.5)	1.07	0.31–3.72	0.905
	Overweight (25-29.9)	1.46	0.83–2.56	0.185
	Obese (≥ 30)	2.06	1.13–3.76	**0.018**
low gait speed (m/s), (≥ 0.8)		4.37	1.75–10.90	**< 0.001**
Low grip strength (kg/cm2), (Female < 16) & (Male < 27)		1.58	1.01–2.48	**0.042**
Calf circumference (Each centimeter increase)		1.02	0.98–1.06	0.214
Mid-Arm circumference (Each centimeter increase)		1.06	1.00–1.12	**0.045**
Waist circumference (Each centimeter increase)		1.02	1.00–1.04	**0.018**
Urinary incontinence (Yes/No)		1.26	0.67–2.39	0.462
Cancer (Yes/No)		2.03	0.59–6.90	0.257
Hearing loss (Yes/No)		1.26	0.63–2.5	0.504
Visual impairment (Yes/No)		1.18	0.72–1.94	0.493
Diabetes mellitus (Yes/No)		1.12	0.68–1.83	0.646
Thyroid (Yes/No)		1.34	0.56–3.19	0.504
Chronic obstructive pulmonary disease (Yes/No)		1.09	0.14–8.43	0.930
Hypertension (Yes/No)		1.08	0.68–1.70	0.729
Congestive heart failure (Yes/No)		1.18	0.61 - 2.28	0.616
cerebrovascular Accident (Yes/No)		1.18	0.75–4.37	0.180
Acute Myocardial Infarction (Yes/No)		0.84	0.30–2.37	0.752
Pain (Yes/No)		1.68	0.91–3.08	0.093
Osteoporosis (Yes/No)		1.75	0.96–3.21	0.067
Osteoarthritis (Yes/No)		1.80	1.10–2.96	**0.019**
Cognitive function (MMSE)	Normal cognitive	Reference group		
	Mild cognitive impairment	1.01	0.60–1.69	0.967
	Dementia	1.81	0.91–3.61	0.088
Depression (PHQ-9)	Normal mood (0–5)	Reference group		
	Mild depression (5–9)	1.21	0.67–2.18	0.508
	Moderate depression (10–14)	2.22	1.18–4.15	**0.013**
	Moderately-severe depression (15–19)	4.57	2.17–9.62	**< 0.001**
	Severe Depressed (20–27)	6.48	2.24–18.73	**< 0.001**
Sleep Quality	Very good	Reference group		
	Relatively good	1.13	0.44–2.90	0.796
	Relatively poor	1.47	0.53–4.03	0.449
	Very poor	4.31	1.22–15.21	**0.023**
Physical activity (Active/ Totally inactive)		0.42	0.26–0.67	**< 0.001**
Impair balance (TUG), (> 12 s)		2.53	1.61–3.97	**< 0.001**
SPPB (Each score increase)		0.52	0.36–0.75	**< 0.001**
Smoking (Yes/No)		0.38	0.12–1.25	0.114
Polypharmacy (Yes/No)		1.74	1.02–2.98	**0.042**
Nutrition status (MNA)	Well nutrition (≥ 24)	Reference group		
	At risk of malnutrition (17–24)	1. 95	1.22–3.11	**< 0.001**
	Malnutrition (≤ 17)	3.85	0.82–18.08	0.087
ADL (Barthel’s index), (Independent/ Dependent)		0.57	0.36–0.91	**0.018**
IADL (Lawton IADL), (Independent/Dependent)		0.84	0.75–0.93	**< 0.001**
Marital status (Married/not married)		0.52	0.31–0.86	**0.011**
Job status	Retired	Reference group		
	Employed	0.27	0.06–1.17	0.081
	Housekeeper	2.18	1.31–3.62	**< 0.001**
	Unemployed	1.06	0.30–3.65	0.921
Education years (Each year increase)		0.97	0.92–1.01	0.209
Living arrangement (Live alone/Live with)		1.23	0.63–2.37	0.537
Wealth quintile	First (Poorest)	Reference group		
	Second	0.89	0.45–1.78	0.761
	Third	0.95	0.48–1.88	0.905
	Fourth	0.54	0.25–1.18	0.124
	Fifth (Richest)	0.91	0.46–1.80	0.807
**Multiple logistic regression model**				
Sex (Male/Female)		0.35	0.20–0.63	**< 0.001**
Depression (PHQ-9 score)	Normal mood (0–5)	Reference group		
	Mild depression (5–9)	0.82	0.44–1.50	0.527
	Moderate depression (10–14)	1.27	0.66–2.47	0.465
	Moderately-severe depression(15–19)	2.96	1.37–6.41	**< 0.001**
	Severe Depression (20–27)	4.28	1.41–12.99	**0.010**
Physical activity ( Active/ Totally inactive)		0.55	0.33–0.90	**0.018**
Impair balance (TUG), (> 12 s)		1.95	1.22–3.13	**< 0.001**

OR = Odds Ratio, CI = Confidence Interval, MMSE = Mini-Mental State Examination, PHQ-9 = Patient Health Questionnaire-9, TUG = Timed up and go, SPPB = Short Performance Physical Battery, MNA = Mini Nutritional Assessment, ADL = Activities of Daily Living, IADL = Instrumental Activities of Daily Living. Significant variables were **bolded**

MMSE scoring:

• Normal cognitive (Illiterate ≥ 19, Primary school ≥ 24, High school ≥ 27, Academic ≥ 28)

• Mild cognitive impairment (Illiterate = ≥ 14 MMSE score < 19, Primary school = ≥ 20 MMSE score < 24, High school = 24 ≥ MMSE score < 27, Academic = ≥ 25 MMSE score < 28)

• Dementia (Illiterate < 14, Primary school < 20, High school < 24, Academic < 25)

In the multiple logistic regression model with backward elimination using a threshold of P < 0.1, male sex (OR = 0.35; 95% CI 0.20 to 0.63), moderate-to-severe depression (odds ratio [OR] = 2.96; 95% confidence interval [CI] 1.37 to 6.41), severe depression (OR = 4.28; 95% CI 1.41 to 12.99), being physically active (OR = 0.55; 95% CI 0.33 to 0.90), and high risk of falls according to the TUG (OR = 1.95; 95% CI 1.22 to 3.13) were found to be associated with fall experiences (Table 2).

Cox proportional regression models were used to analyze the hazard ratios for time to events. In the univariate models, obesity, low grip strength, low gait speed, increased mid-arm and waist circumference, osteoarthritis, moderate-to-severe depression, very poor sleep quality, high risk of falls according to the TUG, polypharmacy, at-risk malnutrition according to MNA, and being a housekeeper were identified as hazards for falling. On the other hand, physical activity, male sex, increased SPPB scores, and increased ADL and IADL scores had a preventive role (Table 3). We considered sex differences and repeated the results according to both sexes. We found that falling in women was related to physical activity (OR = 0.53; 95% CI 0.20 to 0.94), but this association was not found in males. Severe depression was a stronger predictor of falls among men than women (OR = 18.25, 95% CI 1.68 to 198.55 in men vs. 2.88; 95% CI 0.85 to 9.77 in women).

**Table 3:** Associated between fall incidence and its risk factors ( univariate and multiple Cox proportional regression models)

Variables		Hazard ratio	95% CI HR	P-Value
**A univariate Cox proportional regression model**				
Age (Each year increase)		1.02	0.99–1.05	0.124
Sex (Male/Female)		0.28	0.16–0.48	**< 0.001**
Body mass index (kg/m2)	Ideal body (18.5–25)	Reference group		
	Low body (≤ 18.5)	1.04	0.31–3.52	0.938
	Overweight (25-29.9)	1.41	0.81–2.44	0.222
	Obesity (≥ 30)	2.07	1.15–3.70	**0.014**
Low grip strength (kg/cm2) (Female < 16) & (Male < 27)		1.60	1.03–2.47	**0.035**
Low gait speed (m/s), (≥ 0.8)		3.91	1.58–9.68	**< 0.001**
calf circumference (Each centimeter increase)		1.02	0.99–1.06	0.102
Mid-arm circumference (Each centimeter increase)		1.06	1.00–1.12	**0.028**
Waist circumference (Each centimeter increase)		1.02	1.00–1.04	**0.010**
Urinary incontinence (Yes/No)		1.35	0.73–2.49	0.339
Cancer (Yes/No)		2.06	0.65–6.54	0.219
Hearing loss (Yes/No)		1.23	0.63–2.39	0.531
Visual impairment (Yes/No)		1.19	0.73–1.92	0.468
Diabetes mellitus (Yes/No)		1.15	0.71–1.86	0.559
Thyroid (Yes/No)		1.36	0.59–3.14	0.463
Chronic obstructive pulmonary disease (Yes/no)		1.01	0.14–7.31	0.987
Hypertension (Yes/No)		1.02	0.65–1.59	0.915
Congestive heart failure (Yes/No)		1.31	0.69–2.49	0.395
Cerebrovascular accident (Yes/No)		1.63	0.71–3.75	0.248
Acute Myocardial Infarction (Yes/No)		0.86	0.31–2.36	0.778
Pain (Yes/No)		1.56	0.86–2.82	0.142
Osteoporosis (Yes/No)		1.77	0.99–3.15	0.053
Osteoarthritis (Yes/No)		1.93	1.20–3.13	**< 0.001**
Cognitive function (MMSE)	Normal cognitive	Reference group		
	Mild cognitive	1.06	0.64–1.77	0.798
	Dementia	1.56	0.80–3.02	0.185
Depression (PHQ-9 score)	Normal mood (0–5)	Reference group		
	Mild depression (5–9)	1.17	0.65–2.10	0.586
	Moderate depression (10–14)	2.05	1.11–3.76	**0.020**
	Moderately-severe depression (15–19)	4.46	2.24–8.90	**< 0.001**
	Severe Depression (20–27)	5.31	2.06–13.67	**< 0.001**
Sleep Quality	Very good	Reference group		
	Relatively good	1.10	0.44–2.77	0.831
	Relatively poor	1.37	0.51–3.68	0.526
	Very poor	4.08	1.24–13.41	**0.020**
Physical activity (Active/ Totally inactive)		0.44	0.27–0.70	**< 0.001**
Impair balance (TUG), (> 12 s)		2.29	1.47–3.577	**< 0.001**
SPPB (Each score increase)		0.57	0.40–0.80	**< 0.001**
Smoking (Yes/No)		0.41	0.13–1.32	0.139
Polypharmacy (Yes/No)		1.73	1.03–2.91	**0.036**
Nutrition status (MNA)	Well nutrition (≥ 24)	Reference group		
	At risk of malnutrition (17–24)	1.79	1.13–2.81	**0.011**
	Malnutrition (≤ 17)	2.82	0.68–11.61	0.151
ADL (Barthel’s index), (Independent/Dependent)		0.57	0.36 − 0.89	**0.013**
IADL (Lawton IADL), (Independent/Dependent)		0.86	0.78–0.95	**< 0.001**
Marital status (Married/ No married)		0.54	0.33–0.88	**0.014**
Job status	Retire	Reference group		
	Employed	0.29	0.06–1.24	0.096
	Housekeeper	2.09	1.27–3.44	**< 0.001**
	Unemployed	1.06	0.31–3.55	0.922
Education years (Each year increase)		0.97	0.93–1.02	0.290
Living arrangement (Live alone/ live with)		1.18	0.62–2.23	0.603
Wealth Index quintile	First (Poorest)	Reference group		
	Second	0.85	0.43–1.68	0.656
	Third	0.97	0.50–1.86	0.928
	Fourth	0.48	0.22–1.06	0.071
	Fifth (Richest)	0.94	0.49–1.82	0.872
**Multiple Cox proportional regression model**				
Sex (Male/Female)		0.37	0.21–0.64	**< 0.001**
Depression (PHQ-9 score)	Normal mood (0–5)	Reference group		
	Mild depression (5–9)	0.81	0.44–1.47	0.492
	Moderate depression (10–14)	1.31	0.70–2.45	0.382
	Moderately-severe depression (15–19)	2.97	1.47–6.01	**< 0.001**
	Severe Depression (20–27)	3.26	1.24–8.54	**0.016**
Physical activity (Active/ Totally inactive)		0.59	0.36–0.96	**0.034**
Impair balance (TUG), (> 12 s)		1.73	1.10–2.72	**0.017**

h = Hazard ratio, CI = Confidence Interval, MMSE = Mini-Mental State Examination, PHQ-9 = Patient Health Questionnaire-9, TUG = Timed up and go, SPPB = Short Performance Physical Battery, MNA = Mini Nutritional Assessment, ADL = Activities of Daily Living, IADL = Instrumental Activities of Daily Living. Significant variables were **bolded**

MMSE scoring:

• Normal cognitive (Illiterate ≥ 19, Primary school ≥ 24, High school ≥ 27, Academic ≥ 28)

• Mild cognitive impairment (Illiterate = ≥ 14 MMSE score < 19, Primary school = ≥ 20 MMSE score < 24, High school = 24 ≥ MMSE score < 27, Academic = ≥ 25 MMSE score < 28)

• Dementia (Illiterate < 14, Primary school < 20, High school < 24, Academic < 25)

In a multiple Cox proportional regression model similar to the logistic regression model, four variables, namely, sex, depression, physically active condition, and high risk of falls according to the TUG, were significantly related to the time to fall (Table 3). We also calculated the subhazard for death as a competing risk for falls, and the results were not significantly altered (Table 1 Supplementary). Sensitivity analysis was performed to assess the loss to fallow up. In the first scenario, we assumed that all subjects who were lost to follow-up had experienced a fall, and in the other scenario, they did not have this experience. The results of new analyses were within the 95% confidence interval of the main analysis (Tables 2 and 3 Supplementary). Moreover, we compared the characteristics of the lost with non lost participants. There was no difference between the two groups. This increased the probability of completely random missing data (Table 4 Supplementary).

## Discussion

The study aimed to examine the incidence of falls and identify associated risk factors among community-dwelling older adults in the eastern region of Iran. The findings revealed a comparatively low incidence of falls among the participants. Multiple logistic regression and Cox proportional hazard models identified four significant risk factors for falling: sex, depression, physical inactivity, and a high risk of falls based on the TUG test. These results suggest that being female, experiencing moderate-to-severe depression, leading a sedentary lifestyle, and exhibiting a high risk of falls according to the TUG test are associated with an increased likelihood of falling among the studied population of older adults.

In our study, we identified that being active during the week is an important factor in preventing falls among older adults. Active seniors were found to have a 41% lower likelihood of falling than inactive seniors. The World Health Organization (WHO) recommends that older adults engage in moderate physical activity for three or more days per week to prevent falls [29]. Previous research studies confirmed our findings [30–32]. Physical activity plays a significant role in preventing falls and promoting healthy aging by improving mobility, cognition, and independent functioning [33]. Additionally, maintaining an adequate level of physical activity throughout the day has been associated with a 40% reduction in the incidence of fractures among older women. Light household activities have proven effective in enhancing coordination, endurance, and balance [34].

Engaging in regular physical activity over the long term has several benefits for older adults, including improved bone density [35] and muscle mass and strength [36]. It can also reduce the risk of lifestyle-related diseases such as stroke, cardiovascular diseases, high blood pressure, diabetes, and certain cancers by improving maximum aerobic capacity [37, 38] and decreasing oxidative stress and inflammation [39].

Moreover, consistent physical activity throughout the week has a positive effect on cognitive status, dementia prevention, and mental health, including the reduction of symptoms associated with depression and anxiety and overall improvement in mood [40, 41]. All of these factors indirectly contribute to the prevention of falls among older adults. While some studies have reported an inverse or ineffective relationship between physical activity and falling [42], it is generally acknowledged that considering the harms of inactivity and the benefits of physical activity on the body, mind, and spirit, maintaining mobility is preferable to being inactive. The WHO also advises the older adults to engage in regular physical activity [29, 30].

The incidence of falls among community-dwelling older adults during approximately 24 months in this study was slightly over 6%. A systematic review of 104 studies estimated the prevalence of falls among older people at 26.5% globally (27.9% in America, 23.4% in Europe, 25.8% in Asia, 25.4% in Africa, and 34.4% in Oceania) [43]. The fall rate observed in this study is lower than the reported values from other countries. It is worth noting that over 20% of the participants in our study are farmers, and their occupation requires more physical activity, which may indicate better physical health and lifestyle conditions and potentially explain the lower fall rate. Additionally, since the registration of falls was self-reported, it is possible that cases of minor falls without injury were not captured. Pahlevanian attributed the lower rate of falls in Iran compared to other countries to factors such as race, differences in study methods, varying time frames, and different definitions of falls [11]. Moreover, increasing the sample size and conducting studies over multiple years have been identified as two factors contributing to the reduction in the prevalence of falls worldwide [43]. The average age of the participants in this study is 70 years old, which is representative of the mean age of the older population in Iran and could be another factor contributing to the lower fall rate observed.

The study revealed that more than 9% of women and over 2.5% of men experienced falls, indicating that the rate of falling in women was approximately 3.5 times higher than that in men. These findings support previous studies [44]. Several risk factors contribute to higher fall rates in women, including osteoporosis, arthritis, lower balance performance, and prefrailty [45–48]. Gale identified urinary incontinence and frailty as two factors associated with falls in women [49]. The decline in estrogen and muscle and bone mass during menopause further exacerbates the risk of falls in women, as highlighted by Blain (2001), Ning [50], and Lang [51]. Older women also exhibit a higher rate of depression than older men, which can contribute to increased falls among older women, as mentioned in Moreno’s study [52]. Moreover, women who are housewives and more active at home may be more susceptible to domestic accidents, as noted by Sotoudeh [53].

Based on our findings, depression has been identified as one of the risk factors affecting falls in older adults. Previous studies have indicated that depression is indeed a risk factor for falling [54–57]. In the older adults population, the symptoms of depression differ and are often characterized by more pronounced cognitive and functional deficits, as well as increased anxiety [58]. A review conducted in the field of depression in older adults identified several depression-related factors contributing to falls, including cognitive deficits, executive functioning, feeding and sleeping problems, changes in walking patterns, and fear of falling [59]. Furthermore, depression in older adults can lead to decreased appetite, putting them at risk of malnutrition and decreased vitamin D levels. These nutritional deficiencies themselves serve as risk factors for falls [60]. Depression and falling have a two-way relationship. Instances of falling and its associated complications can instill fear of falling, leading to limitations in social participation and social isolation, which in turn contribute to frequent falls [59, 61, 62]. An effective relationship has been found between depression symptoms and various fall risk factors, such as cognitive impairment, slow walking speed, poor balance, slow reaction time, and lack of strength. Each of these factors independently increases the likelihood of falling [59]. Additionally, the use of antidepressant medication may contribute to the increase in falls among depressed older adults due to potential side effects [55, 63]. Considering the high prevalence of depression in the older adults and the comparatively lower awareness and treatment of this condition in this population [64], depression can be considered an important and influential risk factor for falls among aged individuals.

The findings of the present study demonstrate that the use of the TUG test is effective in identifying older adults with a high risk of falling. Moreover, for every 1-second increase in the TUG test, the likelihood of falling increases by 3%. However, the results from various studies regarding the predictive power of TUG have shown conflicting outcomes [65, 66]. Lee recommends implementing TUG as a means to predict falls in community-dwelling older adults [67]. Additionally, in a review exploring various tools for assessing falls in older adults, Park considered 4 out of 26 related tools suitable, including TUG [68]. Despite differing opinions on the use of this test for screening older adults falls, it is recommended by organizations such as The National Institute for Health and Care Excellence (NICE), the American Geriatric Society, and the British Geriatric Society as a routine fall assessment tool [69]. While some studies discussing the use of this test do not reject its utility, they recommend that it should not be relied upon solely to assess fall risk in the older adults [70, 71].

### Limitations and implications

One of the most significant strengths of our study was its prospective longitudinal design, focusing on community-dwelling older adults. We were pleased to have a low participant attrition rate, with less than 1.7% lost to follow-up. This study had a community-dwelling random sample with a relatively high response rate at baseline (more than 65%) and low loss to follow-up, which increases the generalizability of this study.

To the best of our knowledge, this is the first longitudinal study conducted in Iran with such a large sample size and a wide range of variables related to falls.

However, there were certain limitations to consider. Due to executive limitations, we were unable to record the occurrence of falls over short-term periods. Additionally, asking participants about falls multiple times in a short period could potentially make them mentally predisposed to falling, which may introduce bias. In our study, falls were recorded annually, which opens the possibility of recall bias, as individuals might forget to report some incidents. Furthermore, since the assessment of falls relied on self-reporting, it is possible that minor or uncomplicated falls were underestimated and not reported. Another limitation is the lack of information on participants’ previous history of falls.

Overall, while our study had notable strengths, it is important to acknowledge and consider these limitations when interpreting the results.

## Conclusions

The results of the present study found four risk factors associated with falls among community-dwelling older adults: gender, physically active level, depression, and results of timed get up and go. Older women were found to be at a higher risk of falling and should be prioritized for high-risk identification programs and interventions. Depression was a strong risk factor for falling and early detection and treatment of depression should be prioritized to reduce falls risk in older adults. We also found that all physical activity may be protective against falls. Based on these findings, we recommend that older adults adopt an active lifestyle to reduce their risk of falls. The findings of this study have practical implications for screening and prevention programs at the community level. By identifying older adults at high risk of falls based on the identified factors, we can implement targeted interventions and preventive measures effectively.

### Electronic supplementary material

Below is the link to the electronic supplementary material.

Supplementary Material 1

## Data Availability

All data used and analyzed in this study are not available. (We cannot publish public data because Tehran University of Medical Sciences and Birjand University of Medical Sciences, which own the data, do not agree with publishing the data, but if the editors of the journal or someone want it privately should be contacted to Farshad Sharifi; first responsible author.)

## Abbreviations

OR: Odds Ratio
HR: Hazard ratio
CI: Confidence interval
MMSE: Mini-Mental State Examination
PHQ-9: Patient Health Questionnaire-9
TUG: Timed up and go
SPPB: Short Performance Physical Battery
MNA: Mini Nutritional Assessment
ADL: Activities of Daily Living
IADL: Instrumental Activities of Daily Living
BLAS: Birjand longitudinal aging study
WHO: World health organization
