# Supplementary material for: Low physical activity and depression are the prominent predictive factors for falling in older adults: the Birjand Longitudinal Aging Study (BLAS)
Source: BMC Geriatr. 2023 Nov 20;23:758. doi: 10.1186/s12877-023-04469-x (PMC10662773; doi:10.1186/s12877-023-04469-x)
Supplement: Supplementary file 1 — Supplementary Material 1 [file 12877_2023_4469_MOESM1_ESM.docx]

Table 1 Supplementary: Competing risks analysis of sub-hazard ratio for falling according to death fall

| **Variables** | | **Sub hazard** | **95% CI OR SHR** | **P-Value** |
| --- | --- | --- | --- | --- |
| Sex (Male/Female) | | 0.36 | 0.20 - 0.64 | <0.001 |
| Depression (PHQ-9 score) | Normal mood (0-5) | Reference group | | |
|  | Mild depression (5-9) | 0.83 | 0.45 - 1.54 | 0.561 |
|  | Moderate depression (10-14) | 1.31 | 0.69 - 2.49 | 0.406 |
|  | Moderately-severe depression (15-19) | 2.94 | 1.44 - 6.00 | <0.001 |
|  | Severe Depression (20-27) | 3.06 | 1.11 - 8.40 | 0.030 |
| Physical activity (Active/ Totally inactive) | | 0.59 | 0.36 - 0.96 | 0.037 |
| Impair balance (TUG), (>12 s) | | 1.65 | 1.05 - 2.61 | 0.030 |

SHR= Sub hazard, CI= Confidence Interval, TUG= Timed up and go, PHQ-9= Patient Health Questionnaire-9

Table 2 Supplementary: Sensitivity analysis with scenario all of the missed subjects had fall.

| **Variables** | | **Odds. ratio** | **95% CI OR** | **P-Value** |
| --- | --- | --- | --- | --- |
| Sex (Male/Female) | | 0.44 | 0.27 - 0.72 | <0.001 |
| Depression(PHQ-9 score) | Normal mood (0-5) | Reference group | | |
|  | Mild depression (5-9) | 0.85 | 0.50 - 1.44 | 0.554 |
|  | Moderate depression (10-14) | 1.25 | 0.69 - 2.26 | 0.448 |
|  | Moderately-severe depression (15-19) | 2.49 | 1.20 - 5.14 | 0.014 |
|  | Severe Depression (20-27) | 3.89 | 1.39 - 10.84 | <0.001 |
| Physical activity (Active/ Totally inactive) | | 0.57 | 0.37 - 0.88 | 0.013 |
| Impair balance (TUG), (>12 s) | | 1.87 | 1.22 - 2.85 | <0.001 |

OR= Odds Ratio, CI= Confidence Interval, TUG= Timed up and go, PHQ-9= Patient Health Questionnaire-9

Table 3 Supplementary: Sensitivity analysis will consider with all of the missing data hasn’t fall.

| **Variables** | | **Odds. ratio** | **95% CI OR** | **P-Value** |
| --- | --- | --- | --- | --- |
| Sex (Male/Female) | | 0.36 | 0.20 - 0.64 | **<**0.001 |
| Depression (PHQ-9 score) | Normal mood (0-5) | Reference group | | |
|  | Mild depression (5-9) | 0.83 | 0.45 - 1.53 | 0.566 |
|  | Moderate depression (10-14) | 1.27 | 0.65 - 2.46 | 0.473 |
|  | Moderately-severe depression (15-19) | 2.94 | 1.36 - 6.34 | <0.001 |
|  | Severe Depression (20-27) | 3.94 | 1.31 - 11.87 | 0.015 |
| Physical activity (Active/ Totally inactive) | | 0.56 | 0.34 - 0.92 | 0.023 |
| Impair balance (TUG), (>12 s) | | 1.90 | 1.18 - 3.05 | <0.001 |

OR= Odds Ratio, CI= Confidence Interval, TUG= Timed up and go, PHQ-9= Patient Health Questionnaire-9

Table 4 Supplementary: Comparison characteristics of subjects who lost to follow up with subjects continue to participation.

| **variables** | | **Loss to fallow up**  **n (%)** | **Non loss to fallow up**  **n (%)** | **P-Value** |
| --- | --- | --- | --- | --- |
| Age | 60 - 69 years | 13 (56.52) | 777 (57.81) | 0.641 |
|  | 70 - 79 years | 6 (26.09) | 414 (30.80) |  |
|  | 80+ years | 4 (17.39) | 153 (11.38) |  |
| sex | Female | 14 (68.87) | 702 (52.23) | 0.414 |
|  | Male | 9 (39.13) | 642 (47.77) |  |
| Education years | Illiterate | 16 (69.57) | 594 (44.20) | 0.090 |
|  | Primary school | 3 (13.04) | 428 (31.85) |  |
|  | High school | 3 (13.04) | 209 (15.55) |  |
|  | Academic | 1 (4.35) | 113 (8.41) |  |
| Depressed mood (PHQ-9 score) | Normal mood(<10) | 15 (65.22) | 957 (71.47) | 0.510 |
|  | Depressed mood(≥10) | 8 (34.78) | 382 (28.53) |  |
| Physical activity | Totally inactive | 14 (60.87) | 638 (47.54) | 0.201 |
|  | Active | 9 (39.13) | 704 (52.46) |  |
| TUG | Low risk (≤12 s) | 14 (58.33) | 985 (73.29) | 0.106 |
|  | High risk (>12 s) | 10 (41.67) | 359 (26.71) |  |
| SPPB score | Low (0-2) | 1 (4.35) | 43 (3.20) | 0.847 |
|  | Intermediate (3-9) | 8 (34.78) | 407 (30.31) |  |
|  | Robust (10-12) | 14 (60.87) | 893 (66.49) |  |

PHQ-9= Patient Health Questionnaire-9, TUG= Timed up and go, SPPB=Short Performance Physical Battery
